# ASO Author Reflections: Robotic Anatomical Hepatectomy—No Scissors, No Gain?

**DOI:** 10.1245/s10434-025-16961-2

**Published:** 2025-02-04

**Authors:** Emrullah Birgin, Nuh N. Rahbari

**Affiliations:** Department of General and Visceral Surgery, Ulm University Hospital, Ulm, Germany

## Past

The use of the robotic platform for technically demanding procedures such as anatomical segment VIII resection has been controversially debated in the literature owing to limited instruments for parenchymal transection.^1^ Various parenchymal transection techniques were described for robotic liver surgery in the last two decades. These techniques vary mainly by the number of surgeons (one- versus two-surgeon technique) and the type of instruments used for parenchymal transection, i.e., hybrid laparoscopic or robotic devices.^2^ We recently described a unique parenchymal transection technique—the scissor hepatectomy technique—using monopolar scissors and bipolar forceps for meticulous dissection of the liver parenchyma.^3^ In the absence of randomized controlled trials in robotic liver surgery, however, none of the parenchymal transection techniques were compared with each other and proved to be safe for anatomical liver resections.^4^

## Present

In our study, we outlined the technical steps of a robotic anatomical segment VIII resection including trocar positioning and surgical anatomy.^5^ The scissor hepatectomy technique was used for parenchymal transection without additional sealing devices. The procedure was performed in a 75-year-old patient with hepatocellular carcinoma with a background of chronic hepatitis B infection with an uneventful postoperative course and no signs of recurrence at follow-up.

## Future

Our report highlights that the scissor hepatectomy technique enables safe parenchymal transection for complex anatomical liver resections. Future prospective trials are needed to confirm our observations and compare this with other parenchymal transection techniques.
